# Sun-Compass Orientation in Mediterranean Fish Larvae

**DOI:** 10.1371/journal.pone.0135213

**Published:** 2015-08-26

**Authors:** Robin Faillettaz, Agathe Blandin, Claire B. Paris, Philippe Koubbi, Jean-Olivier Irisson

**Affiliations:** 1 Sorbonne Universités, UPMC Univ Paris 06, INSU-CNRS, Laboratoire d’Océanographie de Villefranche, Villefranche-sur-mer, France; 2 Ocean Sciences, Rosenstiel School of Marine and Atmospheric Science, University of Miami, Miami, Florida, United States of America; 3 Sorbonne Universités, UPMC, UMR BOREA 7208, Muséum National d’Histoire Naturelle, Paris, France; Department of Agriculture, AUSTRALIA

## Abstract

Mortality is very high during the pelagic larval phase of fishes but the factors that determine recruitment success remain unclear and hard to predict. Because of their bipartite life history, larvae of coastal species have to head back to the shore at the end of their pelagic episode, to settle. These settlement-stage larvae are known to display strong sensory and motile abilities, but most work has been focused on tropical, insular environments and on the influence of coast-related cues on orientation. In this study we quantified the *in situ* orientation behavior of settlement-stage larvae in a temperate region, with a continuous coast and a dominant along-shore current, and inspected both coast-dependent and independent cues. We tested six species: one Pomacentridae, *Chromis chromis*, and five Sparidae, *Boops boops*, *Diplodus annularis*, *Oblada melanura*, *Spicara smaris* and *Spondyliosoma cantharus*. Over 85% of larvae were highly capable of keeping a bearing, which is comparable to the orientation abilities of tropical species. Sun-related cues influenced the precision of bearing-keeping at individual level. Three species, out of the four tested in sufficient numbers, oriented significantly relative to the sun position. These are the first *in situ* observations demonstrating the use of a sun compass for orientation by wild-caught settlement-stage larvae. This mechanism has potential for large-scale orientation of fish larvae globally.

## Introduction

Dispersal and connectivity are crucial for the persistence and resilience of marine populations [1]. Most demersal fish species disperse during a pelagic larval phase and drifting pelagic eggs and larvae can be transported over hundreds of kilometers by ocean currents [2]. Despite this potential for long distance transport, self-recruitment has been found to be consistently high (often > 30%; e.g., [3, 4]). The behavioral abilities of fish larvae are well developed: at least at settlement-stage, they can sense their environment, swim vertically and horizontally, and orient [5]. Such behaviors are often invoked to explain how larvae can influence their dispersal and favor retention [3, 6, 7]. Vision, hearing, and olfaction can be used by settlement-stage fish larvae to locate a coastal habitat over short distances (meters to kilometers) [8–10]. Larvae likely use different cues for orientation at various stages of development and distances to their settlement habitat [11]. Yet, it is still unclear whether fish larvae are capable of orienting in an oceanic environment where they would have only globally-available cues. Global mechanisms effective for long distance orientation include magnetic or celestial compasses [12], but there is no evidence so far regarding the existence of a magnetic compass in fish larvae. The use of a sun compass was first proposed for Clupeidae [13], then suggested for Pomacentridae larvae [15] and recently observed in laboratory experiments on Apogonidae larvae [16] and *in situ* on reared Pomacentridae larvae [14]. Consistent orientation, through the use of a sun compass for example, could make a difference in dispersal outcome compared to passive advection [17].

While theoretical frameworks exist to include behavior in biophysical dispersal models [18–20], empirical data are still crucially lacking [6, 11, 21]. Furthermore, most studies on larval fish orientation have been conducted in tropical and insular environments: Lizard Island [5, 22, 23] and One Tree Island [10, 16] in the Great Barrier Reef, Australia; Taiwan [24]). Only three studies looked at orientation in (warm) temperate environments [9, 25, 26] and only one was carried out along a continuous shore, although in a subtropical environment and using non-native larvae [14]. This lack of diversity makes the interpretation of such observations difficult and prevents their generalization into a set of orientation rules, which could be implemented in models.

**Fig 1:**
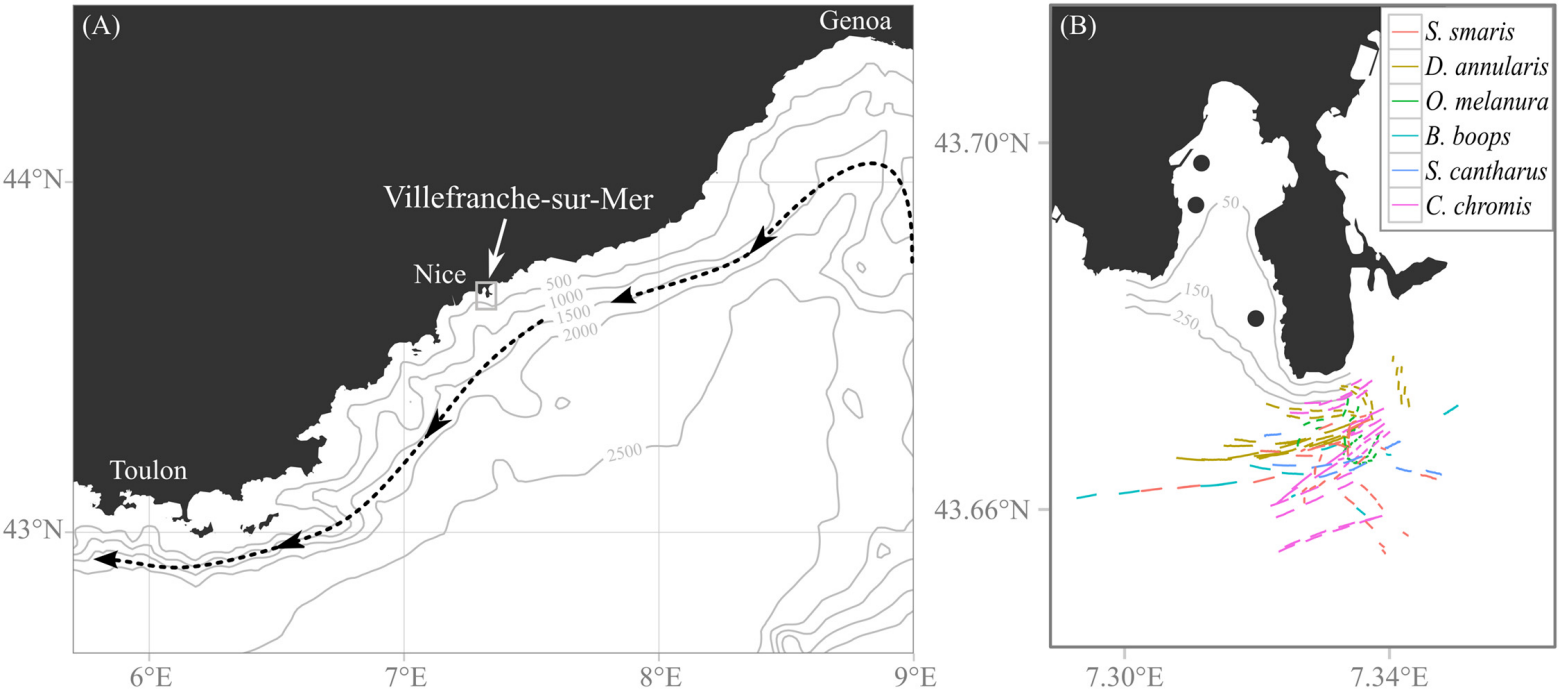
(A) General location of the study area and (B) detailed map of collection sites and deployments. (A) Black arrows represent the average Ligurian current trajectory. Grey lines are isobaths (in meters). The white arrow points the study site (Villefranche-sur-Mer) shown in subplot (B). Nice, Toulon and Genoa are the three main cities in the region. (B) The three black dots represent collection sites of settlement-stage fish larvae. Segments represent the drifting trajectories of the instrument over each 15 min deployment. Grey lines are isobaths (in meters).

The Ligurian coast (North-West Mediterranean Sea) notably differs from insular or tropical environments. The geomorphology is homogeneous; rocky capes alternate with sheltered bays over hundreds of kilometers, from Genova (Italy) to Toulon (France). The continental shelf is extremely narrow, never expanding more than a few hundred meters from the coastline. Settlement and adult habitats are thus constrained to near-shore areas. The Ligurian current is the main oceanographic feature of the region: a strong jet which flows along the coast, between the surface and 200 m depth, at an average speed of 25–35 cm.s^-1^, and creates mesoscale meanders and eddies [27] (Fig 1). No data on larval fish behavior exist in such an environment. The proximity between truly oceanic waters (bottom depth > 1000 m) and the coast makes it very convenient to study the behavior of wild-caught settlement-stage larvae at the end of their pelagic phase, in an oceanic environment.

This study used an *in situ* observation instrument to provide the first data on orientation behavior of Mediterranean settlement-stage fish larvae. We quantified the ability of settlement-stage larvae to keep a bearing and then assessed which environmental variables may influence their orientation. We focused on sun-related variables that could drive orientation over long distances.

## Materials and Methods

### Ethics statement

Samples were collected under permit n°36, delivered on 2014-01-27 by the *Direction interrégionale de la Mer Méditerranée*. The experimental protocol was approved by the University of Miami Institutional Animal Care and Use Committee, under protocol number 11–160 RENEWAL 03. Every effort was made to minimize stress to experimental subjects, which is critical both ethically and for the validity of the behavior observed.

### Larvae collection and handling

Settlement-stage fish larvae (herein “fish larvae”) were caught in Villefranche’s Bay (43.69°N, 7.31°E), which is open to oceanic waters (bottom depth drops to > 300 m at the mouth of the bay; Fig 1) and is known to host rich oceanic plankton communities [28]. The bay may also be a nursery area, thanks to its numerous seagrass beds [29]. Weekly sampling over two years confirmed its suitability for settlement of fish, with large catches of larvae throughout spring and early summer (*pers. data*).

Specimens were collected with CARE light traps, which are effective at capturing fish larvae in the Mediterranean Sea [30]. Moorings were placed at three sites separated by several hundred meters, all with bottom depth > 20 m (Fig 1). Light traps were set one to two hours before sunset and retrieved one hour after sunrise, four days a week between May and July 2014. Fish larvae were sorted visually and kept in 30 L buckets. Back in the laboratory, buckets were placed in a temperature-controlled room at 19°C (close to or slightly lower than seawater temperature measured *in situ*).

**Fig 2:**
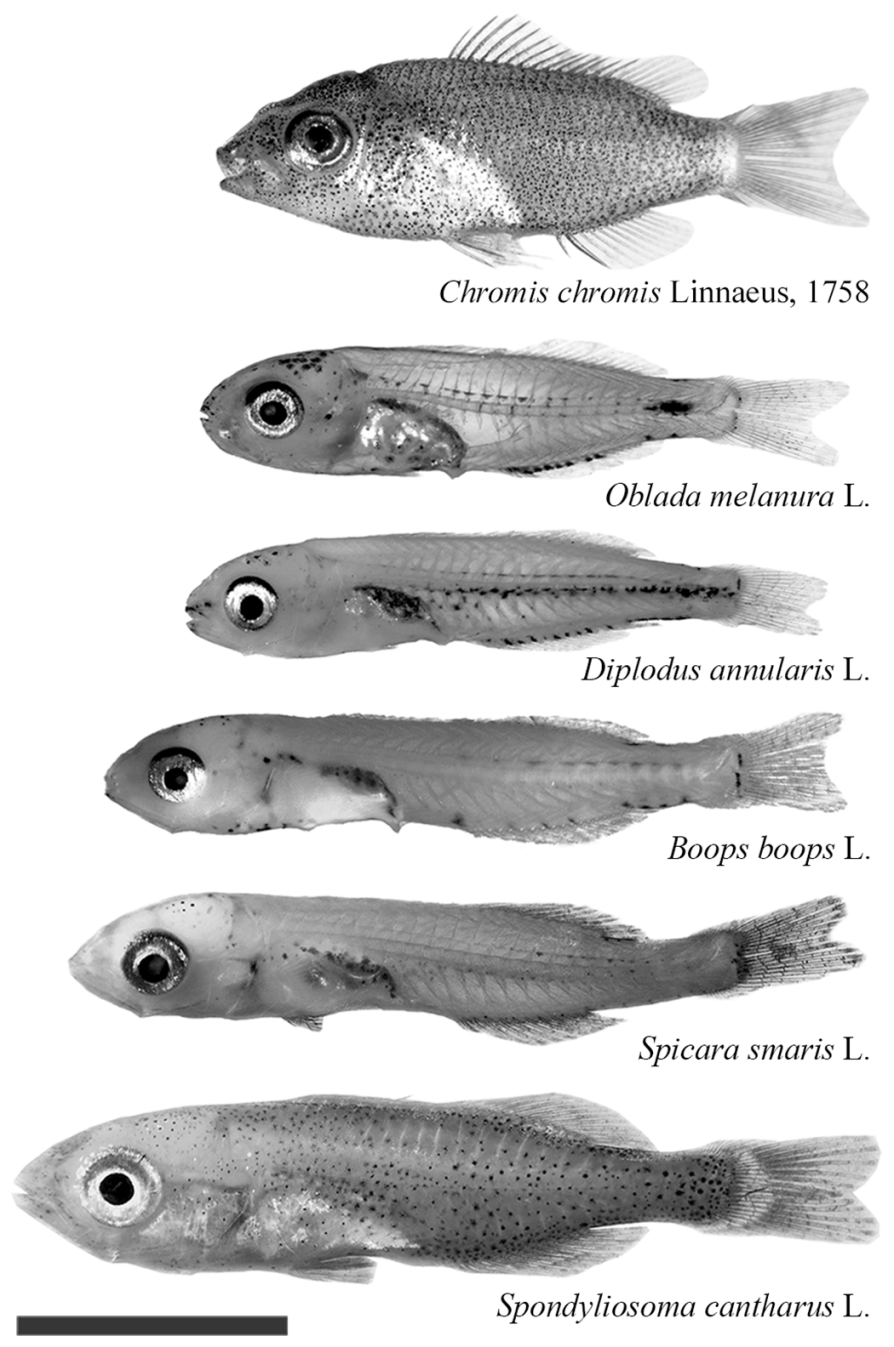
Morphology of the settlement-stage larvae of the six species tested. Size on the figure is proportional to median standard length (scale bar = 5 mm). Species are ordered by increasing size.

Six common species, of ecological and/or commercial interest, were chosen for the tests (Fig 2). *Boops boops*, *Spicara smaris*, and *Spondyliosoma cantharus* were tested between May 7 and 28; *Oblada melanura* and *Diplodus annularis* between June 23 and July 1; and *Chromis chromis* between July 16 and 27.

After a few hours in the lab, fish larvae were taken back offshore to be observed *in situ*. Ninety percent of larvae were tested on the day of capture and 99% within 48h of capture. At the end of the day, larvae were euthanized at -20°C. Within a week, specimens were defrosted, identified to species-level based on [31], and measured (standard length) to the closest 0.1 mm using an image capture software under a microscope (NIS Element 4.11 D).

### DISC description and deployment

The Drifting In Situ Chamber (DISC) is described in [10, 32]. Our configuration is presented in S1 Fig Briefly, it is comprised of a surface buoy that carries a GPS, an acrylic frame, a circular behavioral observation chamber (21 cm diameter x 10 cm height) made of mesh with a 1 mm opening, a GoPro Hero 3+ camera looking upward at the chamber, a custom-made, Arduino-based, numerical compass (plus 3 backup analogical ones) to track its rotation, and a cruciform Microstar Pacific Gyre drogue to lock it into the current.

The DISC is embedded in and drifts with the surrounding water mass, just like larvae would in their natural environment, allowing the larval fish to be observed with no human intervention. To reduce interaction between the structure and environmental cues (sounds, odors, light, etc.), the frame is made of transparent acrylic with approximately the same density as seawater and the observation chamber is made of mesh on the top and the side wall. The cruciform drogue keeps the DISC locked in the current while it interacts with the turbulent flow and rotates slowly. The trajectory of the larva within the chamber informs on its orientation, which will compensate for the DISC rotation if the larva targets a fixed bearing.

Deployments were carried out from a motorboat offshore a cape (Fig 1). Distances to the coast ranged between 200 m and 2800 m. Depth always exceeded 100 m and was most often > 300 m, which prevented visual cues from the bottom. At the start of each deployment, the DISC was pulled to the surface and a fish larva was placed in the behavioral chamber. The DISC was then lowered to the observation depth of 5 m. Each deployment lasted 20 min: 5 min of acclimation and 15 min of observation. Time, GPS position, weather conditions at start and end of DISC deployments, as well as approximate position of boats cruising within 300 m of the instrument were recorded.

### Data processing

DISC data were processed with the open-source software package discusr (https://github.com/jiho/discusr) modified from [33]. The camera produced 5 megapixels images of the fish larva in the chamber at 5 seconds intervals. The position of the larva was recorded on each image by clicking on it through a graphical user interface. Each position was converted to polar coordinates relative to the axis from the center of the chamber to the top of the picture. The angular part of the coordinate was converted to a bearing relative to the North by subtracting the bearing of the top of the picture, recorded by the digital compass. These bearings were the base data to detect cardinal orientation.

In addition to cardinal orientation, the influence of various environmental cues was investigated. The direction and proximity of the coast may influence fish larvae seeking a coastal settlement habitat. In the vicinity of the deployment sites, the underwater landscape is homogeneous and consists of rocky reefs intertwined with seagrass beds. For each deployment, the closest shore point was considered as a potential settlement habitat, and the bearing and distance to it were computed from digitized coastline coordinates.

The sun was considered as a possible directional cue. Its azimuth (bearing of the solar disk) was computed from location and UTC time using [34]. A solar hour index was computed as:

$$\begin{array}{c} \text{solar}\;\text{index}=\frac{t_{\text{deployment}}-t_{\text{sunrise}}}{t_{\text{sunset}}-t_{\text{sunrise}}} \end{array}$$ (1)

where *t*_deployment_ is the mean time of the deployment, and *t*_sunrise_ and *t*_sunset_ are the sunrise and sunset times on the day of the deployment. This solar index ranges from 0 at sunrise to 1 at sunset; 0.5 is the time when the sun is the highest in the sky. It combines the effect of the zenith (angle from the vertical) and azimuth (bearing) of the sun, which are highly interdependent.

Wind, waves, and cloud cover may affect the solar signal. Meteorological data were obtained from Nice airport, located 8 km SW of the sampling zone. They included hourly averages of cloud cover (number of 1/8^th^ of sky occupied by clouds), wind bearing and speed (m.s^-1^).

The drift direction and speed of the DISC were compared with larval orientation direction and strength to confirm that larvae did not simply orient into the current. Finally, the effects of other potentially confounding factors were considered, such as larva size, number of ships cruising by and presence of predators on the pictures.

### Statistical analyses

#### Within-run analyses (at the individual level)

For each deployment, the ability of an individual larva to keep a bearing was tested using the Rayleigh test on the bearings of its positions in the chamber [35]. The test computes a statistic (*r* in [0, 1]), which is a measure of the concentration of the positions of an individual fish larva around its mean bearing (i.e., its *directionality*) and an associated *p*-value.

Non-parametric procedures were used to test for differences in directionality between species, because *r* is bounded in [0, 1] and thus not normally distributed (Kruskal-Wallis test, pairwise Wilcoxon tests with Benjamini-Hochberg correction for multiple testing, and Fligner test). To test the influence of continuous environmental variables on directionality, *r* was logit-transformed and regressed on solar index, wind speed, current speed, distance to the coast and cloud cover using simple linear regression. The logit function is commonly used to transform values from [0, 1] into ]-∞,+∞[ (probabilities for example). The trajectory of the sun in the sky is parabolic and its bearing is easier to assess in the morning and evening, when the sun is low in the sky. This may cause a quadratic rather than linear response to solar index, so both potential relationships were tested. The Shapiro test was used to test the normality of residuals of each significant linear model.

#### Across-run analyses (at the population level)

Within-run analyses only assess the ability to keep a bearing. Across-run analyses are necessary for statistical testing of *orientation* toward a common bearing at the population level. We considered the mean bearings of directional larvae as new data and performed another Rayleigh test. The statistic (*r*) is a measure of the concentration of individual bearings around the mean direction of the population (i.e., orientation *precision*).

To test the influence of directional environmental cues on orientation (direction of the coast, the sun, the wind, the current), we computed the angle between the mean bearing of each fish larva and the bearing of the cue at the time of its deployment. As examples, the resulting angle is 0° if a larva oriented toward the cue and 180° if it oriented away from it. These bearings relative to a cue were also tested with Rayleigh test, to determine the significance of the effect of the cue. The dispersion of the relative bearings around their mean is an estimate of the magnitude of the effect of the cue on orientation (low dispersion = large effect). This is quantified by the value of *r* in the Rayleigh test (large *r* = large effect). When the Rayleigh test was significant for several directional cues, the Wallraff test [35] was used to test for significant differences in dispersion between cues.

#### Remarks on the Rayleigh test

The Rayleigh test is central in this study. Its null hypothesis is randomness in the distribution of bearings. So the alternative is only a “non-random”, typically one-sided, distribution of bearings [35]. But, when the parent distribution of bearings is unimodal (or even better, a von Mises distribution), then a significant Rayleigh test proves not only non-randomness, but also concentration of bearings around the mean direction (i.e., directionality in within-run tests and orientation in across-run tests) [35]. A large sample size (n ≳ 30) is preferable to detect unimodality and allows unimodal distributions to tend towards a von Mises distribution [35]. In within-run analyses, the sample size was 180 (one position every 5 seconds for 15 minutes). We restricted across-run Rayleigh tests to species with more than 30 directional individuals.

Analyses were done in R version 3.1.2 [36] with package circular version 0.4-7.

### Data cleanup

To detect orientation behavior that may be an artifact related to the DISC structure itself (e.g., larvae that oriented relative to the DISC structure, thereby not responding to environmental cues), we identified deployments where the DISC rotated at least 180° and where positions were much more concentrated in the reference of the chamber (*r*_chamber_) than in a cardinal direction (*r*_card_) (*r*_chamber_−*r*_card_ > 0.17, a threshold based on the bimodality of the *r*_chamber_−*r*_card_ distribution). These deployments were then visually inspected to confirm the presence of the artifact; five deployment were rejected on this basis. Three more deployments were rejected because the presence of predators around the instrument visibly affected the position of the fish larva in the chamber. The number of boats cruising in the vicinity increased directionality, although only for *O. melanura* larvae (F = 0.94, R^2^_adj_ = 0.16, *p* = 0.014). Two more deployments were rejected because more than three vessels cruised by.

## Results

A total of 182 fish larvae belonging to six species, in two families, were tested (sample size and body size in Table 1; pictures in Fig 2). All species were considered for comparisons of directionality among species. Orientation analysis was restricted to *C. chromis*, *D. annularis*, *S. smaris*, and *O. melanura* for which ≥ 30 individuals were observed.

**Table 1:** Species tested in this study: taxonomy, sample size (n), and median standard length in mm (median [minimum-maximum]).

**Family**	**Species**	**n**	**Standard length**
Pomacentridae	*Chromis chromis*	48	9.7 mm [8.4–12.6]
Sparidae	*Diplodus annularis*	47	10.1 mm [8.5–11.8]
Sparidae	*Spicara smaris*	37	11.8 mm [9.3–13.9]
Sparidae	*Oblada melanura*	30	10.0 mm [7.9–12.0]
Sparidae	*Boops boops*	11	11.3 mm [9.6–12.1]
Sparidae	*Spondyliosoma cantharus*	9	12.8 mm [11.7–13.9]

### Directionality

**Fig 3:**
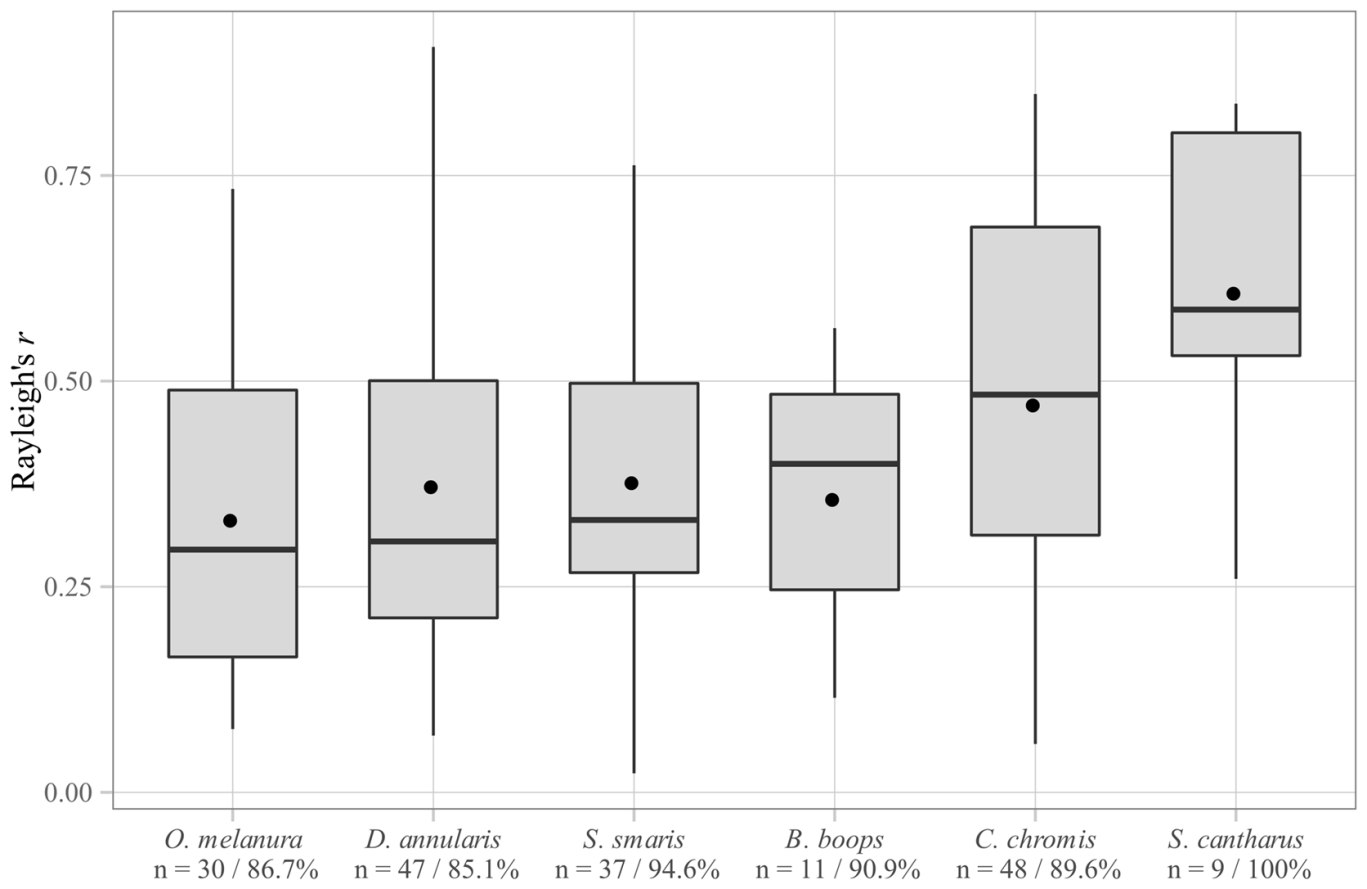
Strength of directionality (Rayleigh’s *r*) of the six species tested. Sample size (n) and proportion of directional larvae (%) are indicated along the x-axis. Standard boxplots (median, interquartile range, and total range) are supplemented with black dots representing mean *r* values.

The vast majority of fish larvae tested were directional in a cardinal reference (within-run Rayleigh test relative to the North, *p* < 0.05), with proportions of directional larvae ranging from 85.1% to 100% among species (Fig 3). Among sparids, *S. cantharus* demonstrated stronger directionality compared to other species (median *r* = 0.59; Fligner, *χ* ^2^ = 4.45, *p* = 0.48; Kruskal-Wallis, *χ* ^2^ = 17.7, *p* = 0.003; pairwise-Wilcoxon, all *p* < 0.05). Directionality was similar in the four other species of this family (*O. melanura* *r* = 0.30, *D. annularis* *r* = 0.31, *S. smaris* *r* = 0.33, *B. boops* *r* = 0.40; pairwise-Wilcoxon, all *p* > 0.05). *C. chromis* was significantly more directional than *O. melanura* and *D. annularis* (pairwise-Wilcoxon, *p* < 0.05) but not significantly different from the other species.

**Fig 4:**
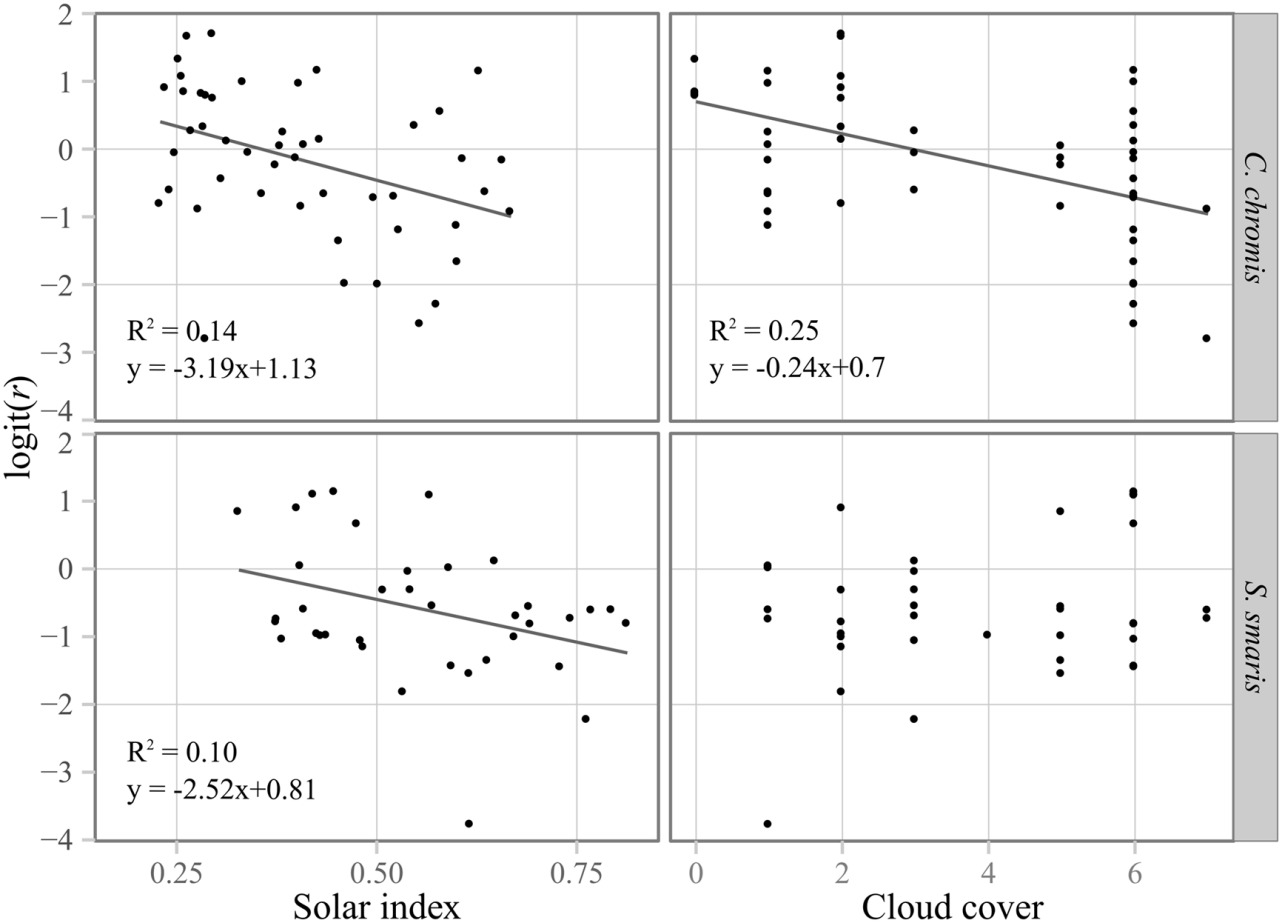
Regressions of directionality (Rayleigh’s *r*) on solar index and cloud cover for *C. chromis* and *S. smaris*. Regression lines are drawn for significant correlations only. Directionality was strong in the morning and decreased linearly along the day. Directionality decreased linearly with cloud cover for *C. chromis*.

Sun-related variables (solar index and cloud cover) most often influenced the directionality of larvae. Directionality decreased throughout the day for both *C. chromis* (F = 1.02, R^2^_adj_ = 0.14, *p* = 0.007) and *S. smaris* (F = 0.94, R^2^_adj_ = 0.10, *p* = 0.034; Fig 4), although the signal was very noisy. The quadratic effects of the solar index were never significant, so the decrease appeared linear. *C. chromis* were also much less directional under cloudier skies (F = 0.95, R^2^_adj_ = 0.25, *p* < 0.001).

In *C. chromis*, directionality appeared stronger when closer to the shore (F = 1.03, R^2^_adj_ = 0.11, *p* = 0.014). However, it could be an indirect effect of the sun, because deployments further away from shore where often done later in the day, when *r* was lower. The relative effects of distance to coast and solar index were discriminated in a linear model of directionality regressed using both variables. Solar index was significant (*p* = 0.035) but distance to coast was not (*p* = 0.084), making solar hour the dominant factor and the effect of distance an artifact, caused by its correlation with solar hour.

Furthermore, in *D. annularis* only, small larvae were more directional than larger ones (F = 1.06, R^2^_adj_ = 0.12, *p* = 0.014). Current or wind speeds never significantly affected directionality, in all species or the pooled assemblage.

### Orientation

**Fig 5:**
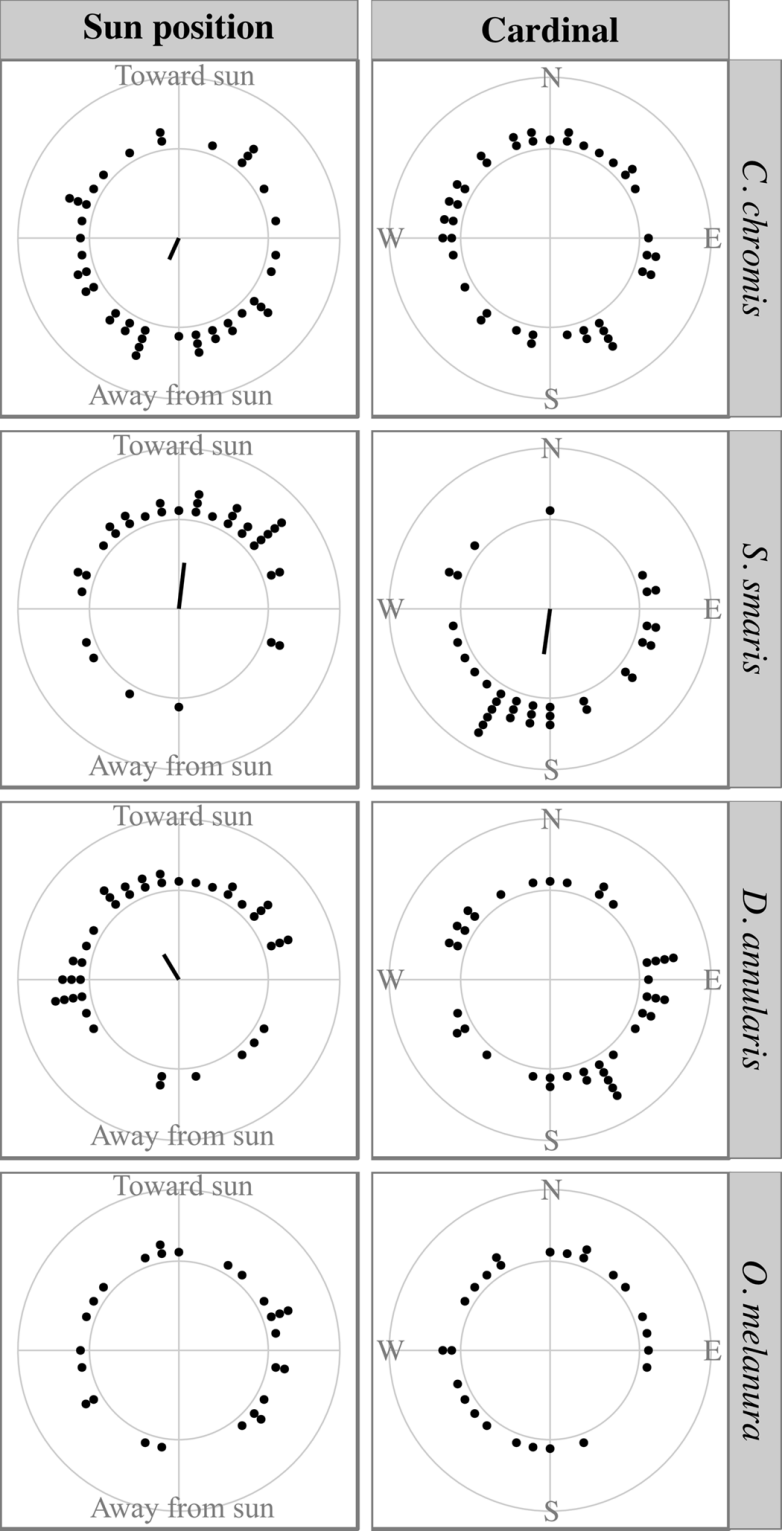
Orientation in cardinal reference and relative to the sun direction. Each dot represents one observation run of 15 min. Mean bearings per-run are binned over 10 degrees. The radius in the middle shows the mean direction of orientation and its length is proportional to the orientation precision (across-run Rayleigh’s *r*).

Only *S. smaris* oriented cardinally, to the south (mean bearing = 188°, *r* = 0.51, *p* < 0.001, Fig 5). For the three other species, the distribution of per-deployment mean bearings was not significantly different from a uniform distribution. Yet, three species significantly oriented relative to the sun direction: *S. smaris* (*r* = 0.52, *p* < 10^-4^), *D. annularis* (*r* = 0.33, *p* = 0.012), and *C. chromis* (*r* = 0.26, *p* = 0.049). They displayed contrasting orientation patterns: the Pomacentridae *C. chromis* oriented away from the sun (mean angle = 207°) while the two Sparidae oriented toward it (*S. smaris*: 7°, *D. annularis*: 329°, Fig 5). The angular dispersion of angles relative to the sun was not significantly different among the three species (Wallraff, all *p* > 0.05).

*O. melanura* exhibited a uniform orientation pattern relative to all tested cues. *C. chromis* and *D. annularis* did not significantly orient relative to any tested cue other than the sun direction. In contrast, *S. smaris* oriented significantly relative to all tested cues (Table 2). These directional cues were partly correlated (S2 Fig): the coast was mostly to the north, the sun was mostly to the south, wind was mostly from the east or south, and current was either from the east or from the west. Still, the orientation relative to the sun direction has the highest *r* value (*r* = 0.52, Table 2), while the sun itself was moving across a 162° range (S2 Fig). However, the angular dispersions were not significantly different between cues (Wallraff, all *p* > 0.05) and cannot be definitive regarding the relative effects of the various cues tested.

**Table 2:** Orientation relative to various cues. For each species: mean angle (°) relative to the cue direction (0° = toward the cue, 180° = away from it); precision of the orientation toward that bearing, quantified by the across-run Rayleigh’s *r*, ranging from 0 (no orientation) to 1 (maximum precision); *p*-value of the across-run Rayleigh test (bold = *p* < 0.05).

	*Chromis chromis*			*Spicara smaris*			*Diplodus annularis*			*Oblada melanura*		
	Bearing	*r*	*p*	Bearing	*r*	*p*	Bearing	*r*	*p*	Bearing	*r*	*p*
North	123°	0.21	0.18	188°	0.51	**< 0.001**	123°	0.21	0.18	123°	0.21	0.18
Sun	204°	0.26	**0.049**	7°	0.52	**< 0.0001**	329°	0.33	**0.012**	52°	0.17	0.49
Coast	13°	0.11	0.62	192°	0.50	**< 0.001**	137°	0.19	0.23	339°	0.20	0.36
Wind	47°	0.17	0.28	249°	0.42	**0.002**	211°	0.19	0.23	357°	0.11	0.76
Current	324°	0.20	0.20	134°	0.32	**0.028**	54°	0.11	0.60	48°	0.28	0.13

## Discussion

### Orientation abilities of fish larvae

These first *in-situ* observations of the orientation behavior of wild-caught Mediterranean fish larvae showed that 89.6% kept a bearing, with a mean individual directionality of *r* = 0.40. This proportion is comparable to what has been observed for the more widely studied tropical fish species (e.g., [10, 15, 23]). Three out of four tested species oriented relative to at least one environmental cue.

For Sparidae specifically, the proportion of directional larvae was higher for all species in our study compared to the two other studies on larvae of this family [25, 26], even though they used a different observation technique which usually yields higher directionality than the DISC [23]. This suggests that Mediterranean Sparidae are at least as capable as their southern-hemisphere counterparts.

Most orientation work has focused on Pomacentridae, using choice chambers [22], underwater following (Leis et *al*. studies summarized in [23]), and DISC [10, 14]. *C. chromis* may be compared with a congeneric species, *Chromis atripectoralis* Welander & Schultz, 1951, which has been extensively studied around Lizard Island, Australia [23]. With the same observation method (the DISC), a similar proportion of larvae were directional (about 90%) but *C. atripectoralis* was always more directional (within-run *r* = 0.67) than *C. chromis* (*r* = 0.48). However, the two species may not use the same cues for orientation, as *C. atripectoralis* showed consistent orientation toward the south-west for almost all combinations of study methods, locations around the island and seasons, while *C. chromis* only oriented relative to the position of the sun in this study.

Within Mediterranean species, *O. melanura*, *D. annularis*, *S. smaris* and *B. boops* had equivalent bearing-keeping abilities at the individual level, while *S. cantharus* and *C. chromis* were both better and not significantly different from each other. Settlement-stage larvae of the first four species are similar morphologically, with small and transparent bodies of almost identical standard length (Fig 2, Table 1), and have pelagic larval durations of 16–18 days [37]. Larvae of *S. cantharus* and *C. chromis* are more muscular, thicker and more pigmented (Fig 2). *S. cantharus* has a longer pelagic larval duration (38 days [37]). *C. chromis* has a pelagic larval duration of about 18 days [37] but larvae hatch from demersal eggs. The noticeable morphological differences at settlement-stage may reflect ontogenetic differences, and the better bearing-keeping abilities of *S. cantharus* and *C. chromis* may reflect a more complete development of their sensory organs. Whatever the mechanism, these results highlighted that orientation ability is likely not general to taxonomic groups, but may be related to morphology and ontogenetic development. Therefore, extrapolation to non-observed species, as is common in modeling purposes, should be made with caution. Gathering more empirical data on larval fish behavior is necessary to make informed parameterization of models or general inferences on community connectivity. Given the results, the settlement-stage larvae of these Mediterranean fish species can be categorized as nekton rather than plankton, as is now accepted for tropical species [5].

### Sun-based orientation in the open ocean

Among the environmental cues tested, sun-related variables such as sun azimuth, solar index and cloud cover were the variables that most often influenced directionality and orientation in this study. The use of celestial cues by fish larvae for orientation has been demonstrated in one tropical species and location, through a laboratory experiment [16]. It has also been suggested *in situ* by the significant effects of time of day [15, 23] and cloud cover [14, 15, 23] on directionality and orientation precision, because time of day and cloud cover affect downwelling light signals and direct view of the sun. With no evidence so far for magnetic orientation in fish larvae, celestial orientation is the only mechanism that could enable large-scale navigation. Indeed, while fish larvae respond to coastal sounds [8], they cannot detect such ambient sounds more than a few hundred meters away from their source [38]. Odors may travel far from their source but, at the scale of centimeter-long larvae, their diffusion is turbulent and extracting directional information from it would be complex [10].

Here, *C. chromis* was less directional under cloudy skies; a result that others have interpreted as indicative of a sun compass [14]. Directionality (within-run *r*) decreased linearly during the day for *C. chromis* and *S. smaris*, which also suggests the use of the sun as a bearing-keeping cue [23]. A quadratic relationship would have been expected, whereby *r* is high at the beginning and end of the day, when the sun is low in the sky and provides a good directional reference, and low around midday, when the azimuth of the sun is more difficult to assess. The linearity of the decrease, more specifically the lack of increase in the afternoon, may be due to increasing nebulosity along the day and to mountains west of the observation location, both of which mask the sun in the evening. In fact, those two variables (cloud cover and solar hour) were the only ones influencing directionality in *S. smaris* and *C. chromis*.

Orientation of *C. chromis* and *D. annularis* was significant only relative to the sun azimuth, not relative to any of the four other cues tested, providing direct evidence of *in situ* sun-based orientation. For *S. smaris*, orientation was significant relative to all tested cues, but the bearings of the cues were correlated and no one cue could be distinguished as significantly more influential than the others. Still, sun-based orientation seems likely for *S. smaris* because its directionality was correlated to the solar index and its orientation precision (across-run *r*) was higher relative to the sun azimuth than to other cues.

Orientation relative to the coast was never significant, except for *S. smaris*, and distance to the coast did not affect directionality, except in *C. chromis* where it was significant but an artifact of the sampling design. So, in the oceanic environment in which larvae were tested (bottom depth > 300 m and distance from coast often > 500 m), it seems they did not react to or could not detect coast-related cues. Without such a point of reference, we hypothesize that larvae used sun-related cues to orient in a fixed direction. Still, the late stages of these species are active swimmers and can travel several kilometers in a few hours (*pers. data*). They could therefore rapidly reach the vicinity of the coast if they swam toward it, and get within the detection range of coast-based cues. The combination of large and small-scale orientation with efficient swimming can drastically affect larval dispersal trajectories and help locate favorable settlement habitats [39].

This study was conducted in summer, around 44°N, where the sun at its zenith is about 20° from the vertical and thus always to the south. Interestingly, the two families tested presented contrasting orientation patterns: Pomacentridae oriented away from the sun, Sparidae oriented towards the sun. Comparing the direction of orientation relative to the sun and the direction of drift in the DISC did not highlight any drift-compensation pattern (e.g., orientation bias towards the east when the DISC drifted towards the west). Both observations suggest that the sun may be used as a reference, a compass for orientation, rather than as an actual goal for navigation. Navigation is the process of ascertaining the position of a goal and following a route to reach it; it is different from (and more complex than) orientation, which is the action of moving based on a compass, not a route. Based on our data, it is difficult to draw a conclusion regarding navigation as larvae were in a space-limited environment and tracked for only 15 minutes. However, the orientation patterns that we observed may be interpreted as larvae calibrating a compass against a universally available cue: the sun’s azimuth. This mechanism was proved possible in settlement-stage fish larvae by a clock-shifting experiment [16]. Furthermore, the sun itself would make little sense as a goal for navigation; its position constantly changes and is not always indicative of the position of a settlement habitat, a food source or other goals relevant for the survival of fish larvae.

Multiple physiological mechanisms could mediate such a sun-based compass, including direct vision of the sun and detection of skylight polarization. Some Pomacentridae can discriminate light polarization and use it for orientation in certain conditions (adults [40]; settlement-stage larvae [14]). For larvae, the vast majority of individuals tested favored direct vision of sun position over polarization axis [14]. Yet, direct vision of the sun from underwater is hampered by clouds or rough seas. In constrast, polarization patterns are equivalent under clear or cloudy skies [41]. Wind speed and sea state never influenced results, and cloud cover affected directionality in *C. chromis* but not in the three Sparidae. So both direct vision and detection of light polarization might have been used by the species observed here to detect the direction of the sun. Determining which mechanism is primarily used for orientation is impossible without additional experiments and direct cue manipulation. Improved understanding of the differences in the utilization of sun-related signals between the two families may explain their opposing orientation relative to sun position.

In the Mediterranean Sea, most fisheries are traditional, coastal, and managed at a small regional scale. The network of marine protected areas is small and not very effective [42]. As a result, the European Union requires that the network is significantly expanded by 2020. Describing fish population connectivity by the simulated dispersal of propagules between protected areas, between fishing grounds, across national boundaries, etc. would be an important step to manage fisheries or plan protected areas at the appropriate scale. However, such models cannot be implemented without knowledge of the pelagic life traits of the simulated larvae. Here, we gathered the first empirical data on larval fish orientation in the Mediterranean Sea. We demonstrated that settlement-stage larvae were capable of keeping a bearing and orienting, similarly to what has been observed in coral reef species. A collection of evidence points toward the sun as an orientation reference, which would enable larvae to orient over large scales during their pelagic phase. To better understand, model and predict connectivity, larval behavior should therefore be considered together with ocean currents, genetic population structure and adult population dynamics; it has received considerably less attention so far.

## Supporting Information

S1 Fig Side view of the DISC during a deployment. (TIFF)

S2 Fig Roses of the bearings of the four environmental cues observed during deployments with ***S. smaris***. (TIFF)

S1 Table Dataset. Bearing-keeping *p*-value (directionality.p.value), directionality (r), larva mean bearing (mean.bearing), cues directions and environmental variables are provided for each deployment.
(CSV)
