# Supplementary figures and images for: Sun-Compass Orientation in Mediterranean Fish Larvae
Source: PLoS One. 2015 Aug 26;10(8):e0135213. doi: 10.1371/journal.pone.0135213 (PMC4550397; doi:10.1371/journal.pone.0135213)

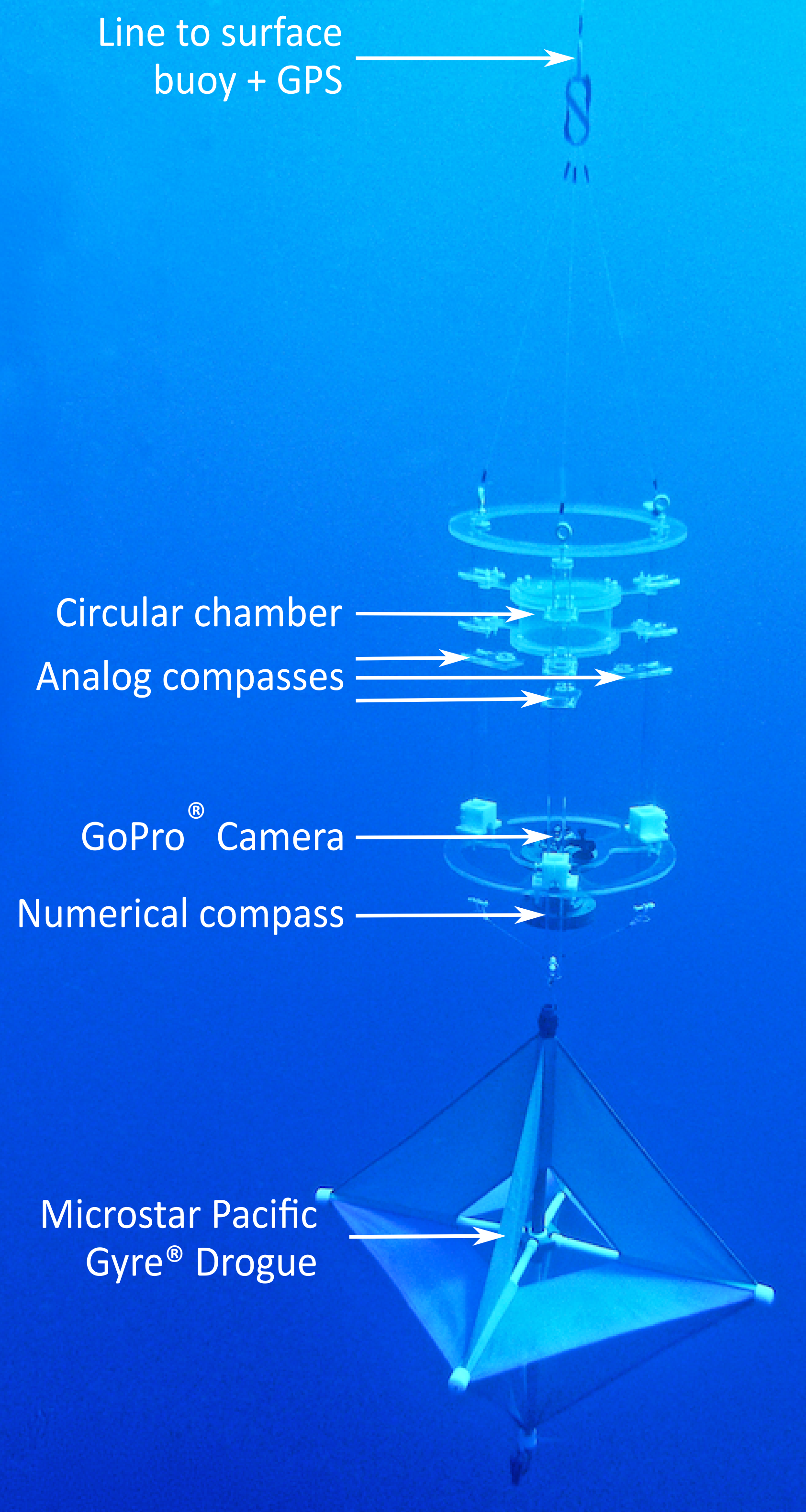

Supplement: S1 Fig — (TIFF) [file pone.0135213.s001.tiff]

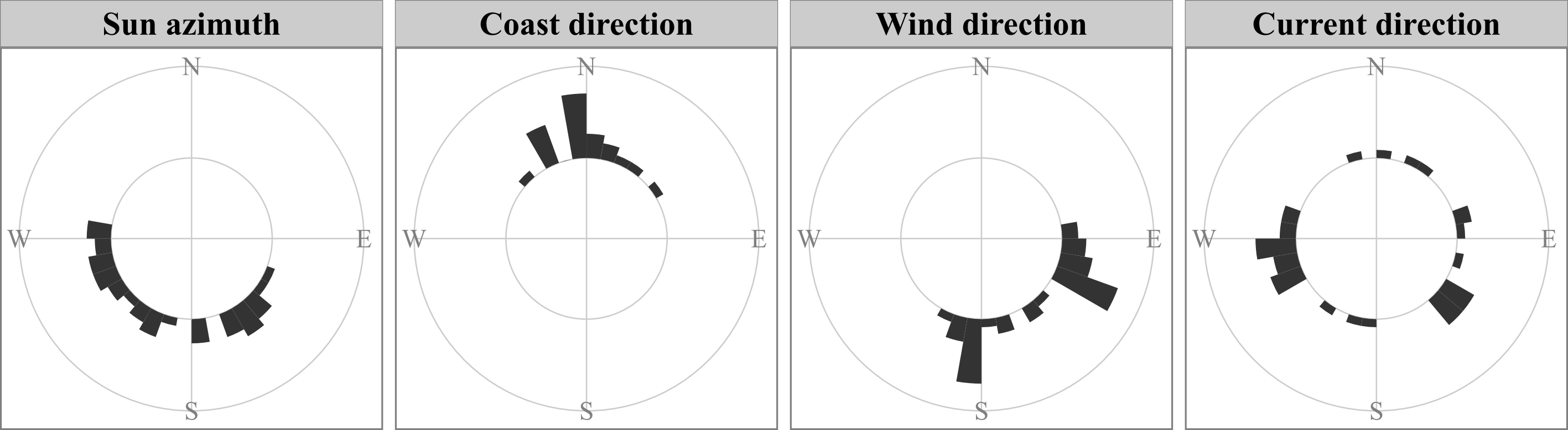

Supplement: S2 Fig — (TIFF) [file pone.0135213.s002.tiff]
